# Bunch mode specific rate corrections for PILATUS3 detectors

**DOI:** 10.1107/S1600577515003288

**Published:** 2015-04-09

**Authors:** P. Trueb, C. Dejoie, M. Kobas, P. Pattison, D. J. Peake, V. Radicci, B. A. Sobott, D. A. Walko, C. Broennimann

**Affiliations:** a DECTRIS Ltd, 5400 Baden, Switzerland; b ETH Zurich, 8093 Zurich, Switzerland; c EPF Lausanne, 1015 Lausanne, Switzerland; d School of Physics, The University of Melbourne, Victoria 3010, Australia; e Argonne National Laboratory, Argonne, IL 60439, USA

**Keywords:** PILATUS detectors, photon counting, count rate correction, Monte Carlo simulation

## Abstract

PILATUS X-ray detectors are in operation at many synchrotron beamlines around the world. This article reports on the characterization of the new PILATUS3 detector generation at high count rates. As for all counting detectors, the measured intensities have to be corrected for the dead-time of the counting mechanism at high photon fluxes. The large number of different bunch modes at these synchrotrons as well as the wide range of detector settings presents a challenge for providing accurate corrections. To avoid the intricate measurement of the count rate behaviour for every bunch mode, a Monte Carlo simulation of the counting mechanism has been implemented, which is able to predict the corrections for arbitrary bunch modes and a wide range of detector settings. This article compares the simulated results with experimental data acquired at different synchrotrons. It is found that the usage of bunch mode specific corrections based on this simulation improves the accuracy of the measured intensities by up to 40% for high photon rates and highly structured bunch modes. For less structured bunch modes, the instant retrigger technology of PILATUS3 detectors substantially reduces the dependency of the rate correction on the bunch mode. The acquired data also demonstrate that the instant retrigger technology allows for data acquisition up to 15 million photons per second per pixel.

## Abstract 2

The count rate behaviour of PILATUS3 detectors has been characterized for seven bunch modes at four different synchrotrons. The instant retrigger technology of the PILATUS3 application-specific integrated circuit is found to reduce the dependency of the required rate correction on the synchrotron bunch mode. The improvement of using bunch mode specific rate corrections based on a Monte Carlo simulation is quantified.

## 1. Introduction   

The single-photon-counting technology of the PILATUS detector series has proved to be very useful for many applications such as X-ray diffraction (Ben-Shem *et al.*, 2011 ▶) or ptychography (Thibault & Menzel, 2013 ▶). The ability to suppress fluorescence radiation with a lower energy threshold and the absence of readout noise substantially improves the data quality in these fields. The counter with a large depth of 20 bits allows for the simultaneous measurement of diffraction spots over an intensity range of six orders of magnitude. The radiation tolerant design of the silicon sensor and the application-specific integrated circuit (ASIC) responsible for the readout minimizes radiation-induced effects from the potentially large amount of deposited X-ray energy.

One of the main challenges for counting detectors is to cope with the high photon fluxes delivered by modern synchrotrons. A dead-time of the order of 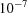
 s leads to perceivable counting losses for photon rates above 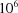 counts per second (cps) and pixel, corresponding to 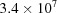 photons per square-millimeter and second for PILATUS detectors. As long as there is a unique relation between the incoming and observed count rate, a count rate correction can be applied to the observed number of counts, thereby extending the usable intensity range. Another approach to extend the high rate capabilities of counting detectors is the implementation of the patented instant retrigger technology as realised in the PILATUS3 readout ASIC (Loeliger *et al.*, 2012 ▶). As explained below, this approach avoids the paralysation of the counting mechanism and increases the viable intensity range by another factor of two.

In the past, the rate correction for the PILATUS detectors was based on an analytical model fitted to experimental data (Kraft *et al.*, 2009 ▶). This simple model provided good corrections but can be improved by considering the dependency of the observed count rate on the energy threshold and the bunch mode of the synchrotron. A first attempt to predict the corrections for different bunch modes based on the mentioned data and using a simple Monte Carlo simulation provided insufficient accuracy. Only including a transistor-level simulation of the PILATUS readout ASIC provided good agreement with experimental data for different synchrotron bunch modes. This work extends the results presented by Trueb *et al.* (2012 ▶) with data for a wider range of synchrotron bunch modes and measurements for the new PILATUS3 detector series.

## 2. The PILATUS detector systems   

**Figure 1:**
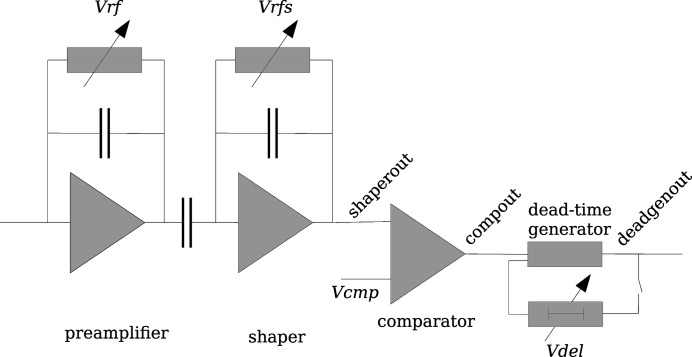
Schematic of the simulated pixel circuit. *Vrf*, *Vrfs*, *Vcmp* and *Vdel* are configurable control voltages. *Vdel* is only relevant in the instant retrigger mode.

**Figure 2:**
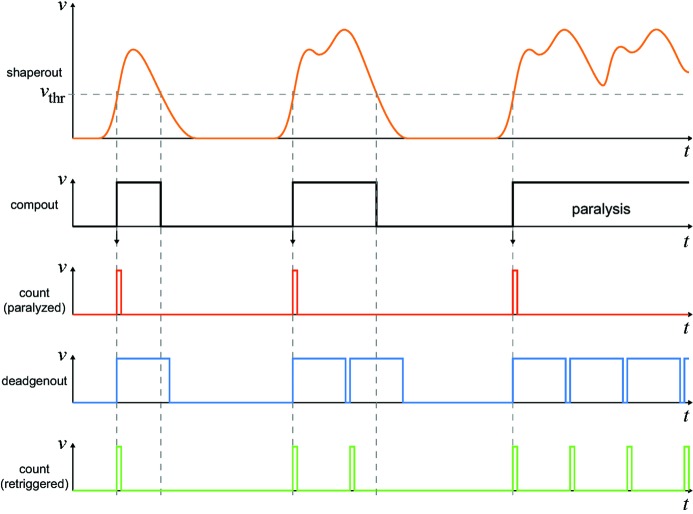
Signal waveforms illustrating the instant retrigger technology of the PILATUS3 ASIC. The observed counts are shown for paralysed (red) and retriggered (green) counting mode [modified from Loeliger *et al.* (2012 ▶)].

As detectors with single-photon-counting capability, PILATUS systems (Broennimann *et al.*, 2006 ▶) belong to the category of hybrid photon counting detectors. The basic building block, a detector module, consists of 16 readout ASICs bump-bonded to a silicon sensor of 320, 450 or 1000 µm thickness. When a photon is absorbed in the sensor, 278 electron–hole pairs are created on average in the silicon for each keV of the photon’s energy. The charge is collected by an applied strong electrical field at the bottom of the sensor where a bump-bond connects each sensor pixel with the pixel cell of the readout ASIC. In the circuit of the readout ASIC (see Fig. 1 ▶), the signal charge is amplified by a charge-sensitive preamplifier, whose gain is controlled by the resistive feedback voltage *Vrf*. After an additional shaping stage, a comparator generates a digital output signal (*compout*) which remains high as long as the shaper output (*shaperout*) exceeds the adjustable threshold voltage *Vcmp* (see Fig. 2 ▶). In the conventional counting mode without instant retrigger technology, the counter is increased for each rising edge of the comparator output. As long as the signal exceeds the threshold level, the pixel is therefore insensitive to incoming photons, leading to complete paralysation at high photon fluxes. In the retrigger mode of the PILATUS3 ASIC, a rising edge of the comparator output triggers a dead-time generator to produce a pulse of configurable length (*deadgenout*). At the end of each pulse, the comparator output is evaluated again and, in the case where it is still high, another pulse of fixed length is generated (Loeliger *et al.*, 2012 ▶). The length of the generated pulse is adjusted by the control voltage, *Vdel*, to be long enough to avoid double counting of single photons at low rates. The counter is increased for each pulse of the dead-time generator. This newly implemented instant retrigger mode avoids the paralysation at high rates. Instead, the observed count rate at high fluxes approaches a value equal to the inverse of the retrigger delay time.

## 3. Simulation   

For arbitrary bunch modes there are no analytical expressions available to compute the observed count rate measured by paralysable or non-paralysable counting detectors. Even if they existed, their usefulness would be limited because readout ASICs such as the one used in PILATUS3 detectors are neither ideally paralysable nor non-paralysable. This can be overcome by the combination of a Monte Carlo simulation with a transistor-level description of the relevant circuits in the readout ASIC. This approach enables computation of the observed count rate as a function of the incoming rate for arbitrary bunch modes and detector settings. Henceforth, the term *incoming rate* refers to the count rate as it would be determined by the detector in the absence of any counting loss due to pulse pile-up.

The determination of the observed rate as a function of the incoming photon rate proceeds as follows: (i) generation of the temporal sequence of the incoming photons; (ii) derivation of the charge collected by a pixel as a function of time; (iii) simulation of the response of the readout ASIC at transistor level.

The detailed implementation of these steps has been discussed in an earlier publication (Trueb *et al.*, 2012 ▶). Using formula (1) of that article, the charge spread in the sensor is estimated to have a value of 7.23 µm for the detector properties presented in Table 1 ▶. It was verified that the simulated results depend only marginally on this value. When using nominal values for the control voltages discussed above, the simulated length of a signal pulse after the shaping stage deviates to some degree from its measured value. In contrast to the previous publication, the overall time scale of the simulation is no longer adjusted to the data by adding a constant offset to the nominal *Vrf* value. Instead, a good agreement is achieved by finding an optimal supply voltage of the analogue part of the readout ASIC. For all simulations in this study, a value of 1.24 V was used for the analogue voltage. For this value the best agreement between simulation and experimental data was found for mid gain settings. By using the same value for all bunch modes and detector settings, the implemented Monte Carlo simulation can indeed be used to predict the rate correction. In other words, the figures shown below do not show a fit of the simulation to the data, but compare the predicted results of the simulation with the experimental data, once the overall time scale of the simulation has been calibrated with data.

## 4. Method of measurement   

The count rate characteristic of the PILATUS3 ASIC has been investigated at four different synchrotrons. A glassy carbon sample was used to scatter the monochromatic beam, so that part of the detector is illuminated with a sufficiently high photon flux. The direct beam has to be stopped to prevent detector damage. The incoming flux is varied by inserting filters of various materials and thicknesses.

The relation between observed and incoming rate is extracted from two measurements, a low-flux image and a high-flux image with a flux ratio of about 100. The low-flux image is used to determine the incoming rate of each pixel. Assuming no counting loss, the average incoming rate is simply given by the number of counts divided by the acquisition time. The acquisition time for both images is chosen to be 50 ms in order to avoid counter overflow at high count rates. The assumption of no counting loss in the low-flux image is met with a precision of a few percent (the precise value depending on the data set and the detector settings) as the count rate in the low-flux image typically does not exceed a few 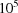
 cps (*cf*. Fig. 5). Knowing the incoming rate in the low rate image as well as the flux ratio between the low and the high rate image, the incoming rate of each pixel in the high rate image can be computed. The observed count rate of a pixel in the high-flux image is calculated as the ratio of the observed number of counts and the known acquisition time. The flux ratio between the low- and high-flux image is determined from data by averaging over all pixels having a count rate of less than 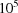
 cps (and therefore negligible count loss) in both images. For the data sets used in the qualitative comparison between data and simulation, several thousands of pixels could be used. As a consequence, the resulting uncertainty on the flux ratio lies below 1%. To obtain the relation between observed and incoming rate (as shown in the Figs. 5 to 8), the observed count rates of pixels with similar incoming rates are averaged and plotted as one data point. The displayed error bars take into account the statistical uncertainties on the number of counts in the low- and the high-flux images as well as the uncertainty on the calculated flux ratio.

Four PILATUS3 detector systems have been used for this project; their key properties are summarized in Table 1 ▶. The detectors have been calibrated by two different methods. For the traditional fixed gain calibration, predefined values for the preamplifier gain are used (low gain: *Vrf* = −0.259 V; mid gain: *Vrf* = −0.159 V) and the threshold voltage is adjusted to match the desired energy threshold. Alternatively, the threshold voltage can be fixed and the preamplifier gain can be varied such that the amplified signal of a photon with an energy of the threshold energy matches the threshold voltage. This second approach is called automatic gain calibration and has the advantage that it allows for the lowest possible gain (and therefore minimum dead-time) for a given energy threshold and noise level.

## 5. Pixel selection   

Pixels are excluded from the analysis

(i) if they lie on the border of an ASIC, or

(ii) if one of adjacent neighbour pixels has a significantly different incoming rate (*e.g.* if they lie on a sharp edge of the beam spot), or

(iii) if the incoming rate of one of their adjacent pixels exceeds some upper value (typically a few 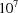
 cps).

**Figure 3:**
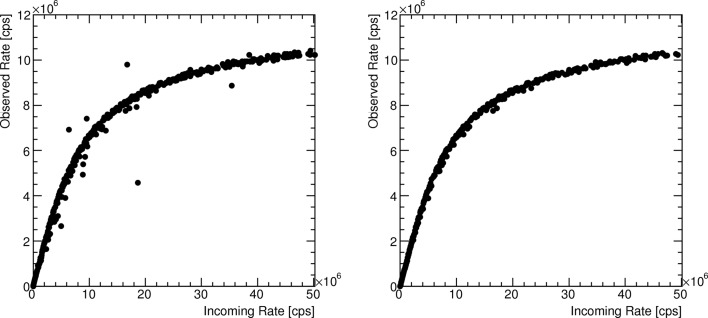
Effect of the pixel selection cuts on the quality of the count rate curves. Shown is the result at low gain settings and an energy threshold of half the X-ray energy of the reference data set before (left) and after (right) applying the cuts. Tight cuts were applied in this data set due to the large number of data points, which removed about one-third of the data points.

The values of the exclusion limits are optimized for each data set individually because the number of pixels and the geometry of the beam spot vary strongly between the different data sets. An example of the effect of the pixel selection on the quality of the count rate curve is shown in Fig. 3 ▶.

## 6. Experimental results   

Before comparing the experimental results with the Monte Carlo simulation, the different data sets shall be briefly presented and a few general results will be highlighted. Data have been collected for seven different bunch modes at four synchrotrons as summarized in Table 2 ▶.

### 6.1. Australian synchrotron   

**Figure 4:**
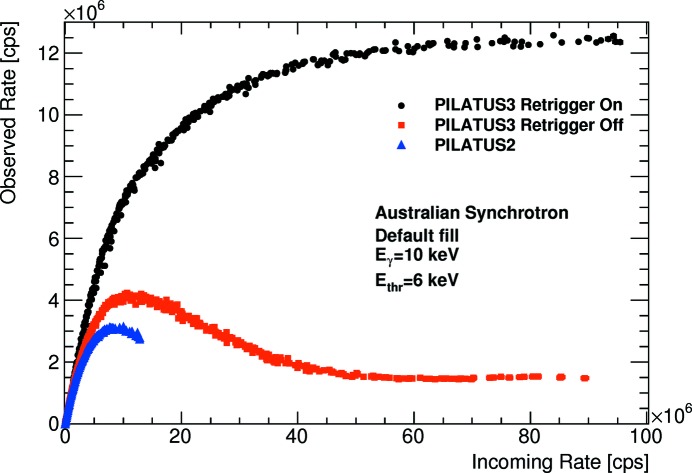
Comparison of the PILATUS3 ASIC, with enabled and disabled retrigger modes, to the PILATUS2 ASIC for the fastest detector settings. The data were taken with the default bunch mode of the Australian Synchrotron.

Two different bunch modes have been investigated at the SAXS/WAXS beamline (Kirby *et al.*, 2013 ▶) at the Australian Synchrotron (AS). The bunch mode labelled AS3 in Table 2 ▶ consists of three electron bunches separated by 240 ns. This mode is very similar to the modes APS24 and ESRF16 which will be discussed below. The data set with the best data quality of this project is the one measured in the default bunch mode AS, whose bunch current has a trapezoidal shape when plotted as a function of time (Sobott *et al.*, 2013 ▶). More than 1000 pixels could be used for the data analysis. Some of them have incoming rates exceeding 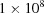
 cps, qualifying this data set as the reference for this article. Although the pixels with the highest rates might not be measured very accurately (as they suffer some count loss even in the low-flux image), these data clearly illustrate the benefits of the instant retrigger technology of the PILATUS3 ASIC. For the fastest detector settings (threshold-to-energy ratio of 0.6, automatic gain calibration), the ASIC is able to count 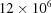
 cps in the retrigger mode, see Fig. 4 ▶. This corresponds to a retrigger time of 83 ns. The measured data show the qualitative behaviour of a non-paralysing detector with no decrease in the observed count rate beyond an incoming rate of 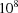
 cps. With disabled retrigger mode, the curve of the PILATUS3 ASIC qualitatively follows the paralysable behaviour of the PILATUS2 ASIC. Above an incoming rate of 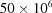
 cps the observed rate amounts to about 1.4 × 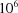
 cps, very close to the synchrotron frequency of 1.38 × 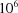
 Hz. The gap of the trapezoidal current seems to be long enough to bring the counter out of paralysation.

**Figure 5:**
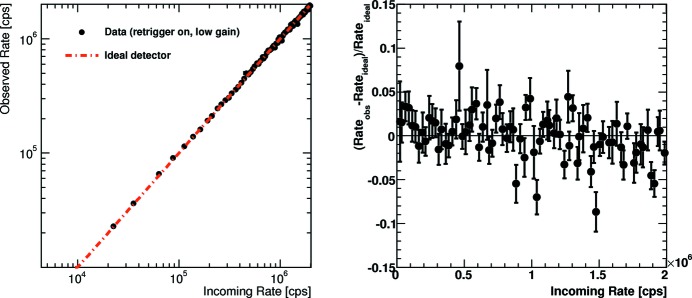
Linearity of the retrigger mode at intermediate count rates without applying a rate correction. Left: comparison with an ideal detector. Right: relative deviation from the ideal detector. The error bars show the experimental uncertainty due to limited Poisson statistics. The measurement was performed with the default bunch mode at the Australian Synchrotron with low gain settings and an energy threshold of half the X-ray energy.

The reference data set can also be used to study the linearity of the detector at medium count rates. While the measurement method is not suitable to investigate the linearity at low rates (the chosen acquisition time results in low statistical precision below a few 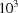
 cps), a closer look at the range 10^4^–10^6^ cps is possible. Fig. 5 ▶ shows this range for low gain settings and a threshold set at half the beam energy for the retrigger mode of the PILATUS3 ASIC. The left-hand plot compares the observed rate with an ideally linear detector. The right-hand plot displays the relative deviation from the ideal detector. The error bars show the experimental uncertainty due to limited Poisson statistics. The expected deviation from the ideal detector due to pile-up becomes apparent above 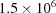
 cps. Below this rate the detector can be operated without any rate correction (for the given settings) with a systematic count rate error of less than 1–2%.

The data from the Australian Synchrotron show no global count rate limit for the PILATUS3 ASIC even for an average incoming rate of 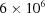
 cps on all of its pixels. Nonetheless, when a whole ASIC is subject to such a high incoming global rate, the observed rate drops slightly compared with an ASIC that is only partly illuminated with high rates. In the extreme case of an average incoming rate of about 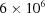
 cps on each pixel, the observed count rate at this incoming rate drops by about 4% for mid gain settings and a threshold of half the X-ray energy. Based on this finding, the number of illuminated pixels was reduced to about 1000 for the reference data set, so that it should not be affected by this effect.

### 6.2. Swiss Light Source   

PILATUS3 detector modules have been characterized at the Optics beamline (Flechsig *et al.*, 2009 ▶) of the Swiss Light Source (SLS). The camshaft mode SLS consists of 390 successively filled bunches separated by 2 ns. The remaining gap of 180 ns is filled with an isolated bunch after 150 ns, containing five times more charge than the other bunches. Overall this bunch mode gives results quite similar to a continuous X-ray beam.

### 6.3. European synchrotron radiation facility   

**Figure 6:**
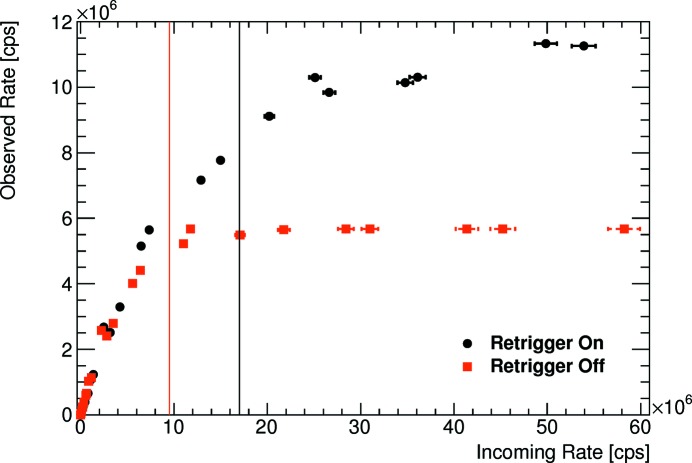
Count characteristic as measured for the 16 bunch mode at ESRF. The red and the black vertical lines are the cutoff rates as derived from the Monte Carlo simulation. Without retrigger mode, the observed rate saturates at 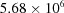
 cps, corresponding to the bunch frequency. After enabling the retrigger mode the saturation value doubles and the rate cutoff increases from 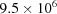 to 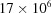
 cps.

Although this data set has very limited statistics and is incomplete (no fixed gain calibration measurements), an important lesson can be learned from it. The bunch mode ESRF16 investigated at the Swiss–Norwegian beamlines at the European Synchrotron Radiation Facility (ESRF) consists of 16 equally spaced bunches separated by 176 ns. For low gain settings this is long enough for the shaper output to fall below the threshold voltage and therefore to release the comparator output before the next bunch arrives. With disabled retrigger mode this results in an asymptotic observed rate of 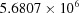
 cps, matching the bunch frequency of 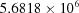
 Hz at the level of 0.02%. When enabling the retrigger mode, the asymptotically observed rate doubles and amounts to 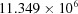
 cps as shown in Fig. 6 ▶. This means that at high rates the dead-time generator always retriggers the comparator output once and two hits are counted for each bunch. In other words, the PILATUS3 ASIC with its instant retrigger technology is able to detect the elongation of the shaper output signal due to pile-up.

### 6.4. Advanced Photon Source   

Three modes have been investigated at the Advanced Photon Source (APS) at beamline 7BM (Kastengren *et al.*, 2012 ▶). The mode APS24 consists of 24 bunches separated by 153 ns, the mode APS of 324 bunches separated by 11.37 ns. The first data set is very similar to AS3 and ESRF16, the second one gives similar results to the modes SLS and AS, which appear to the detector approximately like a continuous beam. The mode APSHybrid consists of eight groups of seven bunches. The seven bunches are separated by 2.4 ns, the group periodicity amounts to 68 ns. After the eight groups, there is a long gap of two times 1594 ns, interrupted by a single bunch in the centre of the gap. This singlet contains up to ten times the charge of any other bunch. As the vast majority of the photons are confined to a short fraction of the synchrotron period, pile-up starts to be noticeable at much lower average incoming rates than for a continuous beam.

## 7. Quantitative comparison between data and simulation   

The PILATUS3 data acquisition software applies the count rate correction only up to some cutoff rate. This cutoff is defined to be the incoming rate at which the slope of the observed rate drops below 0.2. If the observed rate exceeds the corresponding observed cutoff rate, the corrected rate is set to the cutoff rate. This avoids large errors on the corrected rate due to uncertainties on the observed rates. In order to use the results of the Monte Carlo simulation for the rate correction, it has to reproduce the experimental dependency of the observed rate on the incoming rate up to the cutoff rate. Therefore, the following comparison between simulation and data is always restricted to the range up to the cutoff value as determined by the simulated results. For the quantitative comparison between the Monte Carlo simulation and the experimental data, the complete data sets from the AS, SLS and APS are used. A direct comparison of the different bunch modes is possible for the results obtained with the fixed gain calibration.

**Figure 7:**
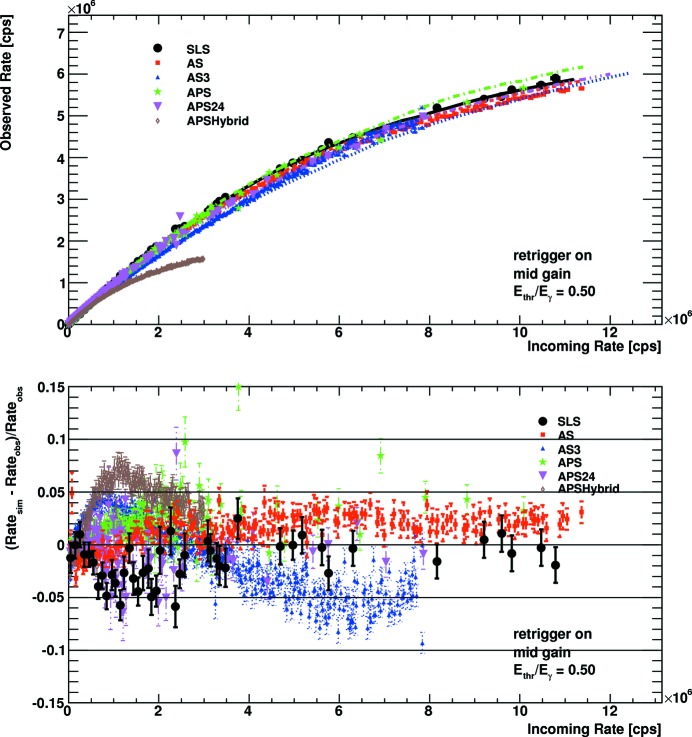
Count rate characteristic (top) and deviation from simulation (bottom) for mid gain settings, an energy threshold of half the X-ray energy, and enabled retrigger mode. In the upper plot the points show the experimental data while the lines are the results of the Monte Carlo simulation.

**Figure 8:**
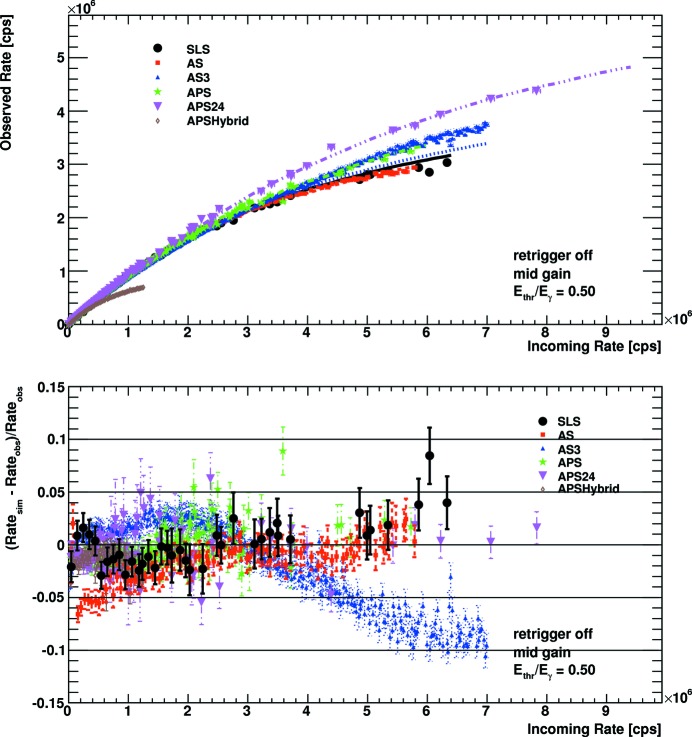
Count rate characteristic (top) and deviation from simulation (bottom) for mid gain settings, an energy threshold of half the X-ray energy, and disabled retrigger mode. In the upper plot the points show the experimental data while the lines are the results of the Monte Carlo simulation.

Fig. 7 ▶ and Fig. 8 ▶ show the count characteristics as well as the deviation between data and simulation for mid gain settings and an energy threshold at half the X-ray energy for enabled and disabled retrigger modes, respectively. Comparing the continuous-like bunch modes for the enabled and the disabled retrigger mode, one finds that for the enabled retrigger mode the cutoff rate is about twice as large. Compared with the mid gain settings of PILATUS2 detectors, the cutoff rate is about four to five times larger. It is evident that the retrigger technology reduces the dependency of the count characteristics on the bunch mode. With the disabled retrigger mode the bunch modes APS24 and AS3, for which the photons hit the detector at repetition rates of about 5 MHz, give the highest observed count rates, followed by the continuous-like modes AS, SLS and APS. When turning on the retrigger mechanism, these differences disappear almost completely. The count behaviour of the detector is then dominated by the retrigger time rather than the properties of the beam. In both cases the simulation reproduces the experimental data well. Up to some outliers, the deviations lie below 5% for the continuous-like modes over the complete range up to the rate cutoff. For the highly structured bunch modes the deviation can amount up to 10%. The interplay of up to three time constants (the bunch spacing, the pulse width after the shaper and the retrigger time) can be quite delicate, so that a small variation in any of them can lead to large changes in the observed rate. As discussed above, some data sets might also be affected by high global count rates.

To quantify the benefit of bunch specific rate corrections, the experimental data were corrected in two different ways, once with the simulated rate curve for a continuous beam and once with the simulated rate curve for the specific bunch mode. Afterwards the relative improvement in reproducing the true incoming rate was computed. The maximum improvements for mid gain settings and an energy threshold at half the X-ray energy are shown in Table 3 ▶. In general the improvements are larger for the disabled retrigger mode, because the retrigger mechanism significantly reduces the dependency on the bunch mode. Only the bunch mode AS3 improves more with the enabled retrigger mode, as the simulation does not optimally reproduce the data with the disabled retrigger mode at high rates. For the continuous-like modes SLS, APS and AS the improvements are up to 10% for the instant retrigger mode and up to 20% without the instant retrigger mode. For the highly structured bunch modes APS24, APSHybrid and AS3 the improvements can be up to 40%.

## 8. Conclusion   

The PILATUS3 ASIC has been characterized for the first time at several synchrotrons with different bunch modes. Its performance is improved in several respects as compared with the PILATUS2 ASIC. No global count rate limit is detected up to incoming rates of about 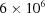 cps on all pixels of an ASIC. For fast detector settings the implemented instant retrigger technology shows no significant count losses up to incoming rates of 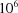 cps even without applying any rate correction. Furthermore, the instant retrigger mode of PILATUS3 detectors is able to provide good data quality up to incoming rates of 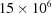
 cps for fast detector settings and makes bunch mode specific corrections dispensable for all but one of the investigated bunch modes. The implemented Monte Carlo simulation reproduces the experimental data with an accuracy of typically 5% for the continuous-like modes and about 10% for highly structured bunch modes. By using these bunch specific rate corrections, the accuracy of the corrected data improves significantly for highly structured bunch modes or when using PILATUS3 detectors with disabled instant retrigger mode.

## Figures and Tables

**Table 1:** Principal properties of the PILATUS detector systems used for this project

Pixel size	172m 172m
Sensor material	Silicon
Sensor thickness	450m
Bias voltage	200V
Counter depth	20bit

**Table 2:** Data set summary

Synchrotron	Bunch mode	Label	Energy (keV)	Maximum incoming rate ( 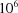 cps)	Analysed pixels	Remarks
SLS	Camshaft	SLS	10.0	36	430	
APS	324 bunches	APS	10.0	10	19000	
APS	24 bunches	APS24	10.0	11	7500	
APS	Hybrid	APSHybrid	10.0	6	19000	
AS	Default mode	AS	10.0	134	1200	Reference data set
AS	3 bunches	AS3	10.0	8	12000	
ESRF	16 bunches	ESRF16	17.5	69	60	Incomplete

**Table 3:** Maximum relative improvement by using bunch mode specific rate corrections The improvement is computed as 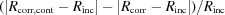, where 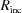 is the incoming rate, 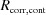 the experimentally observed rate corrected with the simulation for a continuous beam, and 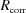 the experimentally observed rate corrected with the simulation for the bunch mode under consideration. The given numbers refer to mid gain settings and a threshold at half the X-ray energy.

Bunch mode	Retrigger on	Retrigger off
SLS	0.07	0.14
APS	0.01	0.04
APS24	0.10	0.24
APSHybrid	0.38	0.40
AS	0.10	0.18
AS3	0.14	0.04

## References

[bb1] Ben-Shem, A., Garreau de Loubresse, N., Melnikov, S., Jenner, L., Yusupova, G. & Yusupov, M. (2011). *Science*, **334**, 1524–1529. 10.1126/science.1212642 22096102

[bb2] Broennimann, Ch., Eikenberry, E. F., Henrich, B., Horisberger, R., Huelsen, G., Pohl, E., Schmitt, B., Schulze-Briese, C., Suzuki, M., Tomizaki, T., Toyokawa, H. & Wagner, A. (2006). *J. Synchrotron Rad.* **13**, 120–130. 10.1107/S0909049505038665 16495612

[bb3] Flechsig, U., Jaggi, A., Spielmann, S., Padmore, H. A. & MacDowell, A. A. (2009). *Nucl. Instrum. Methods Phys. Res. A*, **609**, 281–285.

[bb4] Kastengren, A., Powell, C. F., Arms, D., Dufresne, E. M., Gibson, H. & Wang, J. (2012). *J. Synchrotron Rad.* **19**, 654–657. 10.1107/S0909049512016883 PMC3579593 22713903

[bb5] Kirby, N. M., Mudie, S. T., Hawley, A. M., Cookson, D. J., Mertens, H. D. T., Cowieson, N. & Samardzic-Boban, V. (2013). *J. Appl. Cryst.* **46**, 1670–1680.

[bb6] Kraft, P., Bergamaschi, A., Broennimann, Ch., Dinapoli, R., Eikenberry, E. F., Henrich, B., Johnson, I., Mozzanica, A., Schlepütz, C. M., Willmott, P. R. & Schmitt, B. (2009). *J. Synchrotron Rad.* **16**, 368–375. 10.1107/S0909049509009911 PMC2678015 19395800

[bb7] Loeliger, T., Bronnimann, C., Donath, T., Schneebeli, M., Schnyder, R. & Trub, P. (2012). *Proceedings of the 2012 IEEE Nuclear Science Symposium and Medical Imaging Conference (NSS/MIC)*, pp. 610–615.

[bb9] Sobott, B. A., Broennimann, Ch., Schmitt, B., Trueb, P., Schneebeli, M., Lee, V., Peake, D. J., Elbracht-Leong, S., Schubert, A., Kirby, N., Boland, M. J., Chantler, C. T., Barnea, Z. & Rassool, R. P. (2013). *J. Synchrotron Rad.* **20**, 347–354. 10.1107/S0909049513000411 PMC3943545 23412493

[bb10] Thibault, P. & Menzel, A. (2013). *Nature (London)*, **494**, 68–71. 10.1038/nature11806 23389541

[bb11] Trueb, P., Sobott, B. A., Schnyder, R., Loeliger, T., Schneebeli, M., Kobas, M., Rassool, R. P., Peake, D. J. & Broennimann, C. (2012). *J. Synchrotron Rad.* **19**, 347–351. 10.1107/S0909049512003950 PMC3329955 22514168

